# “DIY” Silica Nanoparticles: Exploring the Scope of a Simplified Synthetic Procedure and Absorbance-Based Diameter Measurements

**DOI:** 10.3390/ma13143088

**Published:** 2020-07-10

**Authors:** Łukasz Tabisz, Jerzy Stanek, Bogusława Łęska

**Affiliations:** Faculty of Chemistry, Adam Mickiewicz University in Poznań, Uniwersytetu Poznańskiego 8, 61-614 Poznań, Poland; stanek@amu.edu.pl (J.S.); bogunial@amu.edu.pl (B.Ł.)

**Keywords:** Stöber silica, nanoparticles, size determination, UV-Vis spectrophotometry, method refinement

## Abstract

In this study, the classical Stöber silica synthesis protocol was used to test the limits of simplification in the preparation and size determination of nanoparticles. The scope of three-ingredient, one-pot synthesis was established in conditions of regular 96% and 99.8% ethanol as solvent, with aqueous ammonia as the only source of base and water. Particles with diameters in the 15–400 nm range can be reliably obtained with this straightforward approach, and the direct relationship between the size and the product of concentrations of water and ammonia is evidenced. Furthermore, the idea of a linear approximation for Mie scattering in particular conditions is discussed, using experimental data and theoretical calculations. A simple, fast method for particle size determination utilizing a UV-Vis spectrophotometer—an easily accessible instrument—is explained, and shows a level of error (<0.5 SD) that can be acceptable for less rigorous laboratory use of nanoparticles or serve as a quick means for testing the influence of minor alterations to known synthetic protocols. This work aims to show that nanoparticle synthesis can (and should) become a regular occurrence, even in non-specialized labs, facilitating research into their new applications and inspiring outside-the-box solutions, while discussing the drawbacks of a more relaxed synthetic regimen.

## 1. Introduction

In recent years, the chemistry behind the synthesis of new materials has come to rival the most long-standing and expansive fields of organic, inorganic, and analytical chemistry. As is true with most sciences, however, these disciplines no longer have well-defined boundaries. In particular, as we study ever smaller particles—venturing into the nanometer range—we can now directly inspect (and question) the blurred distinction between molecules and materials [1,2,3]. Of particular importance to the present paper is the observation that while cutting-edge research in the chemistry of materials continues, its previous accomplishments are of tremendous use to other branches of chemistry, e.g., chromatography, catalysis, and the study of interfacial phenomena [4,5,6]. However, more innovative ideas call for a solution that must be individually tailored and made, not simply bought—a task that many non-material chemists believe is beyond their reach. This notion should be challenged, and simplified methods of synthesis and analysis of (nano)materials need to be developed and disseminated, in parallel with sophisticated research requiring uncommon equipment.

This “backtracking” can yield unexpected insights and solutions [7,8,9], of value even to those far more specialized in the field. The original Stöber process has recently seen its 50th anniversary and remains one of the cornerstones of sol-gel chemistry [10].

Much research has drawn on the idea of the bottom-up synthesis of nanoparticles [11,12,13], and even the original method is regularly revisited, highlighting its accessibility and ease of modification [14,15,16]. It can still be used as a reliable source of silica particles, with a very narrow size distribution, in the range of 10–800 nanometers [16,17] (larger are also reported [18,19]). The appeal of a simple, one-pot preparation of such uniform (and such small) spheres is not limited to didactics. While the ease of preparation of Stöber silica cannot be disputed, a multitude of factors still influence the resulting size and homogeneity of material, as shown by a number of papers [14,16,17]. In practice, in large-scale experiments with many samples, it is quite common to encounter “botched” runs, and untrained hands, of course, exacerbate the risk. Therefore, during our work, we have encountered the rising need for a streamlined synthesis and size-determination procedure, in order to quickly eliminate errors in nanoparticle preparation. As for synthesis the most important factor is the ammonia/water/tetraethylorthosilicate (TEOS) ratio, we decided to reduce the number of reagents and swap the ethanolic ammonia solution (commonly encountered in published procedures) for an aqueous one, which is perhaps one of the most rudimentary laboratory commodities, along with setting the TEOS concentration to a fixed value. More importantly, following the observation of a seemingly linear increase in the perceived turbidity of post-reaction mixtures, we aimed to establish if, and in what circumstances, simple UV-Vis spectrophotometer measurements could be used for the routine and straightforward determination of Stöber nanosilica (and, by analogy, possibly other nanoparticle) sizes. The theory of light scattering is well-established and for homogenous spheres in particular it has been described by Mie as a solution to Maxwell’s equations [20,21,22]. Many authors have tackled the problem of using turbidity measurements—in series, using approximations or different wavelengths [23,24,25]—for the determination of the average size of material particles, which is in itself a crucial and dynamic field of research [26,27]. A large body of work is also devoted to the limitations of that methodology when it comes to giving proper information about the size distribution in samples [28,29]. The idea of using a modified UV-Vis spectrophotometer, in combination with Mie theory-based computer calculations, is also not new [25,30,31]. To the best of our knowledge, however, bridging the gap between that rigorous and complicated model with feasible measurements using an instrument primarily designed for absorption determinations, while retaining the simplicity of that determination (using a convenient linear approximation), has not yet been reported.

## 2. Materials and Methods

### 2.1. Reagents

Ethanol (EtOH, 96% and 99.8%) and ammonia solution (NH_3_, 25% in water) were obtained from Avantor Performance Materials POCH, Poland S.A., Gliwice, Poland. Tetraethylorthosilicate (TEOS) and hydrofluoric acid (HF, 48% in water) were purchased from Merck (Kenilworth, NJ, United States). All chemicals were used without further purification but were either freshly opened (ammonia and ethanol) for each series of experiments or stored under argon (TEOS) in between them.

### 2.2. Equipment and Software

The size distribution of particles was analyzed using transmission electron microscopy (JEM 1200-EX, JEOL Co., Tokyo, Japan). For each sample, at least four micrographs from different sections were taken, in at least two different magnifications, and analyzed with ImageJ (Fiji) free software (ImageJ 2.0.0-rc-69/1.52p, fiji.sc). The samples were randomly assigned a number (scrambled) in order to perform blinded data analysis. Theoretical calculations were run using Maple software (Maple 16, Maplesoft, a division of Waterloo Maple Inc., Waterloo, Ontario).

Visual light absorption/transmission measurements were taken on three different spectrophotometers, as explained in detail in the following sections: Jenway 6305 UV-Vis spectrophotometer, Jenway 6400 Vis scanning spectrophotometer (Cole-Palmer Ltd., Vernon Hills, IL, USA), and Agilent 8453 Diode Array spectrophotometer (Agilent Technologies, Santa Clara, CA, USA).

### 2.3. Preparation of Silica Nanoparticles

A total of 44 nanoparticle samples were obtained in three separate experiments. The first set (18 samples) utilized standard 96% ethanol as the solvent, while the second and third used 99.8% ethanol (18 and 8 samples, respectively). The synthetic conditions were informed by previous reports [10,17,19], but specifically restricted due to our objectives. Only three reagents were used, with the volume of TEOS set to a constant 4% (*v/v*) and the amount of aqueous NH_3_ (25%) being the main variable (3–26% *v/v*); ethanol made up the rest of the reaction mixture. Silica syntheses were performed in 15 cm^3^ plastic screw-cap tubes, washed with 2% HF, and rinsed with deionized water.

In a typical experiment, 9.6 cm^3^ of ethanol-ammonia solution (with varying proportions of NH_3_) was directly prepared in a tube equipped with a small magnetic stirring bar, closed, and placed in a small rack on top of a magnetic stirrer (set to 1000 rpm). After 2–3 min, the TEOS addition (0.4 cm^3^) was made, and the tubes were closed for the time of the experiment, i.e., 72 h. The temperature in the room was kept at 22 ± 1 °C through the use of an air conditioning system.

### 2.4. Spectrophotometric Measurements

Measurements (as absorption/attenuation) were made for samples taken directly from post-reaction mixtures and sufficiently diluted as needed. For all samples, at least three different dilutions (prepared by the addition of small aliquots of ethanol) were analyzed. Suspensions of the smallest nanoparticles could—and had to—be measured with no-to-minimal dilution, while the largest required dilution factors of at least 1:20. The standard wavelength used was 400 nm, but for the third series, measurements were taken at 320, 400, and 500 nm; they were also repeated using three separate spectrophotometers.

## 3. Results and Discussion

### 3.1. Scope of Synthesis

**Figure 1:**
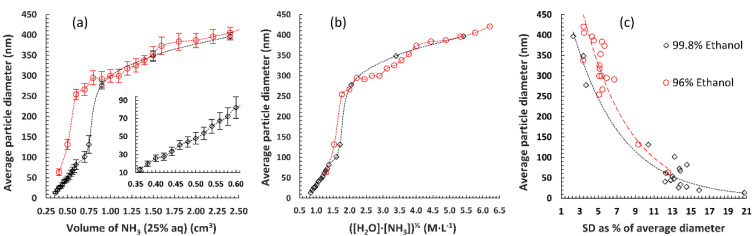
Results of nanosilica synthesis using aqueous ammonia solution and different grades of ethanol as the solvent. (**a**) Average diameter as a function of NH_3_ volume (per 10 cm^3^ of reaction mixture, 0.4 cm^3^ of tetraethylorthosilicate (TEOS)) with error bars used to denote the standard deviation (SD) in size; expanded section shows changes in the lower particle size range, obtained by varying the ammonia volume by 20 μL steps. (**b**) Superimposition of both experimental curves after taking into account the real amount of water in the system and calculating the square root of the product of concentrations of both reagents. (**c**) Relationship between %SD and the average size of obtained nanoparticles, showing a logarithmic decrease in %SD with rising particle size.

The first two series of nanoparticles were obtained using 96% and 99.8% ethanol as solvent, respectively. They were aimed, principally, at estimating the size range of Stöber silica that is attainable with the simplified, three-ingredient procedure. The smallest diameter of particles was 63 nm for the first and 13 nm for the second series, while both experiments gave nanoparticles of about 400 nm in diameter with the highest studied concentration of ammonia (Figure 1; details are available in Table S1 in the Supplementary Material).

It quickly became evident that using 99.8% ethanol is beneficial, both from the point of view of obtainable silica sizes and their mean diameter fully adhering to a monotonic trend (and thus being easily predictable). With 96% EtOH in the higher diameter range, however, random fluctuations of the average particle size were common, even when large parts of this series were actually repeated. This was also reflected in standard deviations, which became consistently larger (compared to those in samples prepared using absolute alcohol) above 200 nm (Figure 1c). For both series, the curve depicting the relationship between the mean particle diameter and amount of ammonia had a shape reminiscent of an acid-base titration curve (although obviously shifted). Therefore, even with absolute alcohol, it is more difficult to obtain particles with a very specific diameter in the range of 150–250 nm than smaller or larger in size (Figure 1a).

**Figure 2:**
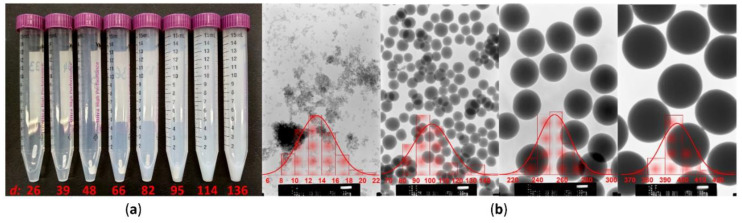
Comparison of different silica nanoparticles prepared using the simplified methodology. (**a**) Turbidity of post-reaction mixtures containing an identical volume fraction of particles with an increasing mean diameter *d*. (**b**) Transmission electron microscope (TEM) imagesof silica spheres of various sizes, acquired using the same magnification to highlight their differences; average diameter distribution histograms are shown in red. All values are given in nanometers.

Comparing the average diameters with the square root of the product of calculated NH_3_ and H_2_O concentrations gave nearly superposed curves (Figure 1b). This is in agreement with the notion that the limiting factor in the seeding of silica particles is actually the hydroxide anion concentration [32,33,34]. It is worth noting that the second experimental series, obviously focusing on the smaller particle sizes, was successful in pinpointing the minimum amount of ammonia necessary for silica synthesis. Volumes below 3.6% (as seen by the last data point in Figure 1a, in the expanded section) did not result in well-defined, smaller spheres, but instead gave a mixture of silica powder and a waxy residue—probably partially hydrolyzed and crosslinked TEOS (extending the reaction time to 6 days did not significantly alter this outcome). Taking into account that with both grades of EtOH the reaction failed at similar dilutions, it can be assumed that (with 25% aqueous ammonia) the NH_3_ concentration is actually the more limiting factor, compared to water. Finally, it should be highlighted that smaller-sized nanoparticles obtained with this simplified methodology, while still near-monodisperse (Figure 2), were less uniform than some examples previously reported in the literature [35].

### 3.2. Turbidity and Reliable Spectrophotometric Measurements

The turbidity in post-reaction mixtures was visible to the naked eye for nearly all samples, and even with the lowest volumes of ammonia added (resulting in a characteristic “egg yolk in water” colloidal appearance), it was still possible to organize them by eye according to the resulting particle size (Figure 2). Therefore it seemed plausible that spectrophotometric measurements could at least yield a “calibration curve”, i.e., a graphical display of the relationship between the diameter and attenuation of the sample (translated into transmittance, translated in turn into the more methodically correct turbidity value), which would allow for future estimations, made directly using samples from post-reaction mixtures. However, while the ammonia volume–particle diameter relationship was nearly linear in the lower range of silica sizes (Figure 1a), another trend was observed with the calculated specific turbidity: samples around 100 nm and larger seemed to be arranged around a straight line, while smaller samples fell on a more exponential curve. It was also determined at this point that while measurements of particles up to 150 nm can be performed without too much difficulty, bigger particles show a gradually rising tendency to sediment; this could result in very large errors. Therefore, care must be taken during the sampling step (suspension must be uniform, vigorously shaken just prior), dilution, and actual measurement, which should be repeated as needed to ensure reproducibility. The largest particles that could be reliably measured this way (i.e., directly in post-reaction medium) were just below 350 nm in diameter.

### 3.3. Theoretical Considerations

To examine whether a linear approximation for the relationship between the specific turbidity *τ_sp_* and particle diameter *d* is at all supported by the theory of light scattering, we used the rigorous Mie solution as a base for calculating the scattering coefficients (*Q_scat_*). The details of the procedure (as Maple code, with comments) are available as Code S1 in the Supplementary Material; only the crucial steps will be presented herein, followed by a discussion of the specific case of Stöber silica and the simplifications stemming from a narrow diameter range, near-monodispersity, and synthetic methodology.

As described by previous authors [21,22,23,36,37,38], the turbidity *τ* of a sample, analyzed at incident wavelength *λ*_0_ (Equation (1) [25,38]), can be linked with *Q_scat_* (Equation (2) [36]) by means of the number concentration *N* (Equation (3) [25,28]). Further simplification by the use of volume fraction *φ* (Equation (4) [28]) finally yields Equation (5) [28], describing the relationship between the specific turbidity and particle diameter:

(1) $$\tau_{\lambda_{0}}=\frac{1}{l}\times ln\frac{I_{0}}{I}=\frac{\ln\left(10\right)}{l}\times A,$$

(2) $$Q_{scat}=\frac{2}{\alpha^{2}}\overset{n_{max}}{\underset{n=1}{\sum }}\left(2n+1\right)\left({\left|a_{n}\right|}^{2}+{\left|b_{n}\right|}^{2}\right)\;,$$

(3) $$\tau_{\lambda_{0}}=N\times \frac{\pi \times d^{2}}{4}\times Q_{scat}\;,$$

(4) $$\varphi =N\times \frac{\pi \times d^{3}}{6}\;,$$

(5) $$\frac{\tau_{\lambda_{0}}}{\varphi }=\tau_{sp}=\frac{3\times Q_{scat}}{2\times d}\;,$$

where *l* is the path length of the beam of light and *A* is the measured attenuation. Definitions of complex functions *a* and *b* can be found in reference [36] and in Code S1 in the Supplementary Material. The scattering coefficient *Q_scat_* is calculated using the dimensionless parameter *α*, which is dependent on *d* through the relationship described by Equation (6) [28,36]:

(6) $$\alpha =\frac{\pi }{\lambda_{m}}\times d\;.$$

The wavelength of light in the medium, *λ_m_*, is related to incident wavelength *λ*_0_ by the simple Equation (7) [36]:

(7) $$\lambda_{m}=\frac{\lambda_{0}}{\eta_{m}}.$$

The refractive index of the medium *η_m_* (together with the refractive index of particle *η_p_*) is also required for the calculation of *m = η_p_/η_m_*, which is another value included in the calculation of *Q_scat_*_._ Therefore, for the precise theoretical determination of a single scattering coefficient value, the following must be known:

- *d*-diameter of particles suspended in the medium;
- *λ*_0_-wavelength of incident light;
- *η_m_*-refractive index of the medium at that wavelength;
- *η_p_*-refractive index of particles at a given wavelength.

**Figure 3:**
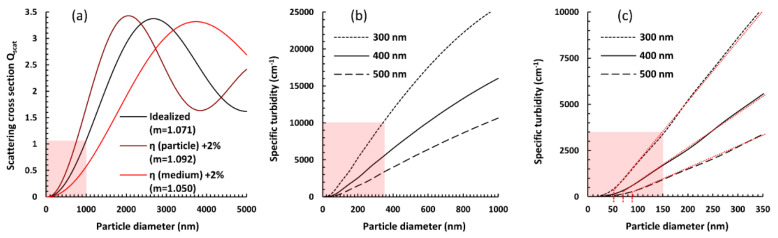
Theory of light scattering on silica particles suspended in ethanol. Pale red sections indicate fragments of the graphs most significant for a further discussion of the specific case described in the paper, which are expanded on in further panels. (**a**) Scattering coefficient *Q_scat_* directly calculated using Mie theory; also shown is the large impact of even small deviations in refractive indices on the value and shape of the function. (**b**) Differences in the plots describing the relationship between the particle diameter and specific turbidity, showing that the apparent linearity in the size range is dependent on the wavelength used (expanded shadowed section of (a), with *Q_scat_* translated into *τ_sp_*). (**c**) Expanded lower-diameter section (from (**b**)), with red dotted lines showing a possible linear approximation and the cut-off point shifting steadily towards smaller particles with a shorter wavelength of incident light. The shadowing in the last panel shows the diameter section crucial for pinpointing this linearity cut-off point, which inspired the third experimental series of nanoparticle syntheses.

Setting aside for the moment the non-ideal, experimental conditions, we tested the theory using values of *η* for fused silica and ethanol acquired from reference [39]. It should be noted, however, that even small deviations in any of the refractive indices result in very large differences in *Q_scat_* (Figure 3a).

While the dependence of the Mie scattering coefficient (or scattering cross section) on the particle size is famously complicated and usually presents as a damped sine wave with another set of oscillations visible in the main curve (see Figure 3 in reference [40]), a relatively small difference in the particle and medium refractive indices (m < 1.1, as is the case for silica and ethanol) results in a flattened plot, with these secondary waves being barely noticeable (Figure 3a). Furthermore, after we translated values of *Q_scat_* into *τ_sp_*, the linearity of its relationship with the particle size (up to 400–800 nm, depending on the wavelength) became evident (Figure 3b), corroborating our experimental results. The “cut-off point” for linearity could indeed be placed near 100 nm. Notably, however, modeling shorter wavelengths of incident light resulted in the range of linearity shifting towards smaller particle sizes, suggesting that employing such wavelengths could expand the applicability of the simplified turbidimetric method (Figure 3c).

Having established the theoretical background for a possible linear approximation method of particle size determination, a third experimental series was tested, aimed at nanosilicas with sizes in the range highlighted in Figure 3c. This series was premeditated as a test for compatibility between theory and the experiment, as well as an examination of possible sources of error.

### 3.4. Reconciling Theoretical and Experimental Results

**Figure 4:**
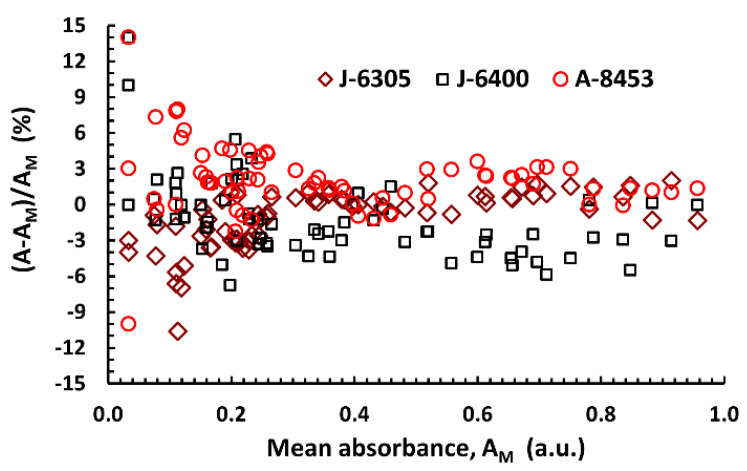
Graphical analysis of all corresponding absorbance measurements (at 320, 400, and 500 nm) taken using different spectrophotometers (see the Materials and Methods section for details), showing the random scattering of points around the mean value. Lack of ordering suggests that instrumental correction factors in turbidimetric measurements are either negligible for small particle sizes or extremely similar, despite different device specifications. However, it is apparent that measurements at attenuation values higher than 0.2 a.u. should always be preferred when the turbidity of a sample (and not “true” absorbance/attenuation) is being evaluated.

It has been thoroughly established that due to small-angle scattering of light (i.e., in the near-forward direction), spectrophotometric measurements must be corrected to account for that phenomenon when used for turbidimetric determinations [30,31,38]. However, it has also been stated that for particles smaller than 500 nm, neither mathematical corrections nor adjustments to the measuring device itself may be needed [25]. To test this assumption in our particular case, attenuation of samples produced in the third experimental series (generally in good agreement with previous materials obtained using 99.8% ethanol), again directly taken from post-reaction mixtures, was measured using three separate spectrophotometers, in addition to different dilutions. No distinctive multiplicative factor was found when analyzing the differences in attenuation values (Figure 4); this could either mean that it is constant, i.e., independent of the construction specifications of the spectrophotometer, or that, indeed, due to small particle diameters, it can be safely omitted in our particular case.

Furthermore, the precision (mean average deviation) of an average triplicate measurement on one device was similar to that obtained using three separate devices (±0.0114 vs. ±0.0069 a.u.). Nevertheless, a simple multiplicative correction factor—uniform for all wavelengths—was found to be necessary to resolve the difference between theory and the experiment, with the latter consistently returning 1.7× larger values of *τ_sp_* for a given particle diameter than anticipated using idealized assumptions and equations. While, at first, this may seem a rather large coefficient, a careful analysis of possible, singular sources of error (Table 1) demonstrates quite clearly that—when compounded—these factors are the most probable (and certainly realistic) cause of its value. Among these factors, miscalculations in (wavelength-dependent) refractive indices (due to the medium being an inconstant mixture of ammonia, water, and ethanol of varying proportions, and the silica particles not being calcinated or even fully hydrolyzed on the surface), along with noticeable SD of silica sizes, are definitely the chief suspects for sources of discrepancies. 

**Figure 5:**
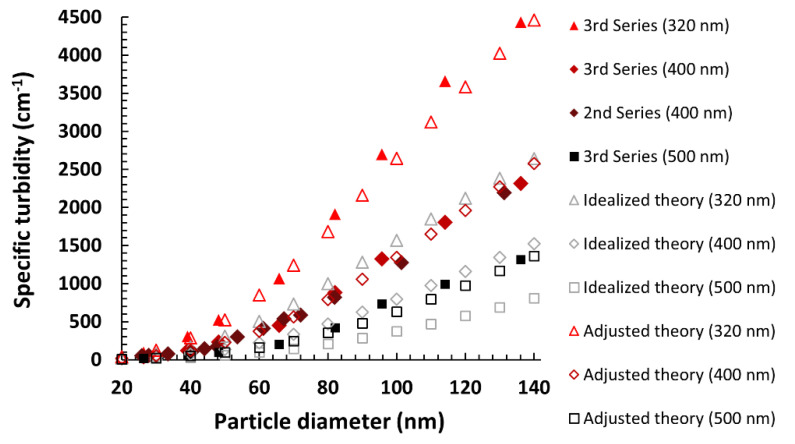
Superimposition of theoretical (idealized and adjusted with a multiplicative factor of 1.69) and experimental plots for *τ_sp_* = f(*d*) showing good agreement and a near-linear relationship for diameters over 60 nm.

Importantly, however, after application of the experimental correction factor, a linear approximation for the determination of the silica nanoparticle mean size turned out to be highly achievable for a 50–350 nm diameter range (Figure 5).

**Figure 6:**
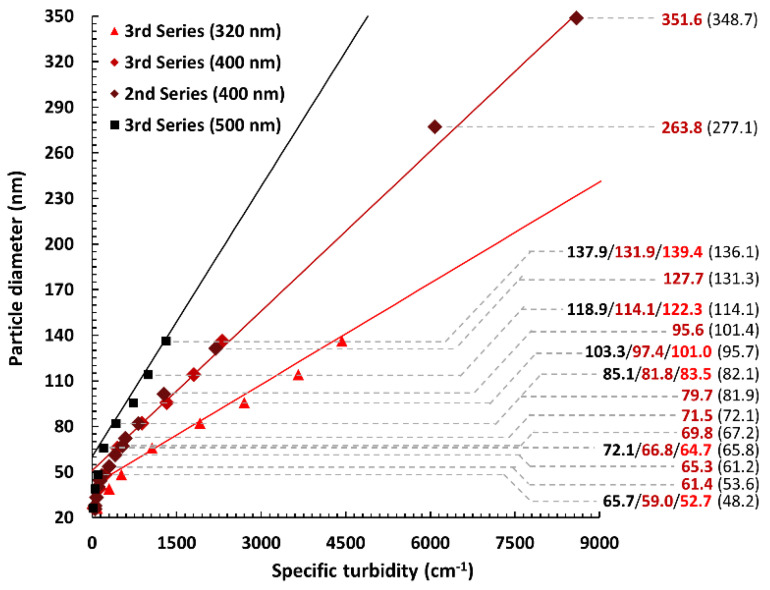
The linear approximation models (for three wavelengths of visual light) for the calculation of the mean particle diameter of Stöber nanosilica samples obtained using the simplified methodology. Numbers on the right show values calculated using functions derived for 500/400/320 nm wavelengths and those obtained from TEM (in brackets). All approximations yield good results down to particle sizes of ~100 nm, those for 400 and 320 nm are applicable down to ~65 nm and even slightly below 50 nm the 320 nm model still returns tolerable (error < 10%, <1 SD) values, in accordance with previous theoretical investigations.

As a final proof of concept of the postulated simplified method for silica particle mean size determination, we utilized the flexibility of a linear function to derive straightforward equations, relating the attenuation of a sample to the diameter of particles suspended in it (in our particular experimental conditions). Reversed plots are presented in Figure 6, along with calculated and true (established by TEM) values of the mean diameter for particles prepared in the second and third experimental series. The following Equations (8)–(12) describe the full procedure of using the linear models, starting with the calculation of the specific turbidity from absorption data:

(8) $$\tau_{sp}=\frac{\tau_{\lambda_{0}}}{\varphi }=\frac{\ln\left(10\right)\times A}{l\times \varphi_{0}\times \left(\frac{V_{s}}{V_{s}+V_{d}}\right)}=414.1\times \frac{V_{s}+V_{d}}{V_{s}}\times A,$$

where *V_s_* and *V_d_* denote the volume of sample (taken directly from the post-reaction mixture) and diluent (ethanol), respectively, mixed before measuring the absorbance/attenuation (i.e., dilution factor), and *φ*_0_ is the (constant) volume fraction of particles in the post-reaction mixture, calculated from experimental conditions. In the particular case discussed in this paper, the parameter 414.1 cm^−1^ is obtained after supplying all known values.

Obtained *τ_sp_* values can be directly used to calculate mean silica particle diameters using the linear approximation models:

(9) $$d=\tau_{sp}\times a+b,$$

(10) $$\lambda_{0}=320\;nm\;:\;a=0.0222,\;b=41.0813,$$

(11) $$\lambda_{0}=400\;nm\;:\;a=0.0350,\;b=50.0989,$$

(12) $$\lambda_{0}=500\;nm\;:\;a=0.0593,\;b=60.1116.$$

As can be seen from Figure 6, it is recommended that a sample is analyzed using at least two wavelengths (as well as in two or three dilutions). While longer wavelengths seem to be advantageous with larger particles, the reverse is definitely true. For particles smaller than 65 nm in diameter, using wavelengths longer than 320 nm (with the aim of utilizing the linear model) is strongly discouraged.

## 4. Conclusions

In this study, the scope of a simplified, three-ingredient synthetic methodology for the preparation of Stöber silica nanoparticles was established. Using a constant volume fraction of TEOS (4%) and 25% aqueous solution of ammonia as a combined source of base and water (3.6–24%), well-defined particles with mean diameters in the range of 13–400 nm and a narrow size distribution can be conveniently obtained. The benefits of using 99.8% ethanol (as compared to regular 96% solution) were also identified: easier control over mean particle sizes and, on average, slightly lower standard deviations in their values. 

Importantly, after the translation of added reagents’ volumes into the product of concentration of water (including water from aqueous ethanol) and ammonia, both synthetic curves (displaying the particle diameter as a function of concentration) aligned very well with regard to the slope and lower and upper “asymptotic” regions. 

This, again, reinforces the notion of hydroxide anions playing a crucial role in TEOS hydrolysis and condensation. At the same time, it was observed that a similar amount of ammonia (~3.6% *v/v*, which corresponds to 0.48 M) was the lower limit for successful silica preparation, regardless of the ethanol grade used (and, therefore, the water concentration). While not the focus of the present study, the partially hydrolized organosilicon products recovered from syntheses near this concentration could prove an interesting research subject, both from the point of view of their molecular structure, and as a way to better understand the mechanism of silica condensation (or sol-gel processes in general). 

Furthermore, following experimental observations and calculations (using Mie theory for the scattering of light), it was postulated that—in the specific case of small silica nanoparticles suspended in ethanol—it is possible to develop linear approximations for the relationship between the specific turbidity (calculated from attenuation measurements taken using a regular spectrophotometer) and the diameter of those particles. As a proof of concept, three such linear models were developed and their results compared with TEM-based mean diameter measurements. In most cases, errors were smaller than 3% and almost never exceeded 0.5 SD; however, the method is constrained in the lower (<100 nm) and higher (>350 nm) diameter range. In the smaller particle regimen, only shorter wavelengths of light (i.e., the 320 nm model) return acceptable results, due to the linearity “cut-off point” in Mie scattering-based theory. With larger particles, sedimentation becomes a problem, precluding samples directly taken from post-reaction mixtures to be analyzed and complicating the procedure beyond the originally intended scope. Nevertheless, the observations and ideas discussed in this paper can be of use to non-specialized laboratories in need of a straightforward synthesis and analysis method for silica nanoparticles. The flexibility offered by linear approximations, taken together with the prevalence of silica-based nanomaterials, can result in many alterations to the discussed protocol with retention of the original simplicity. A range of modifications: in size (at least up to 800 nm), volume fraction (i.e., experimental conditions), and even the chemistry of the particle and medium (so long as the ratio of their refractive indices does not become much higher than 1.1) should be perfectly possible without violating a linear model after sufficient adjustments. In particular, research concerning the optimization of co-condensation reaction parameters (e.g., ratio of organosilicon compounds and temperature) could greatly benefit from adaptation of the proposed technique, while using it as an auxiliary tool for studying the differences between freshly obtained materials and those after washing and drying/calcination is also an interesting future research avenue.

## Figures and Tables

**Table 1:** Analysis of single variable changes that would be necessary for explaining the experimental correction factor.

Variable	Idealized Value ^1^	Corrected Value ^2^	% Change Needed
η_p_	1.470	1.498	+1.90%
η_m_	1.370	1.347	−1.68%
φ_0_^3^	0.00556	0.00945	+70.0%
(R) ^4^·A	1.0·A	0.6·A	−40.0%
d (SD%) ^5^	0% ^5^	55% ^5^	− ^5^

^1^ Values used in initial theoretical calculations [39,41]; ^2^ value needed for the best agreement of theoretical and experimental plots of *τ_sp_* = f(*d*); ^3^ particle volume fraction calculated from experimental conditions assuming a 100% yield of SiO_2_ from TEOS and a 1.95 g/cm^3^ density of particles; ^4^ correction factor for measured attenuation, e.g., related to small-angle scattering influences (although this would rather result in overestimations); ^5^ standard deviation in particle diameter as the % of mean diameter; while, for theoretical calculations, this factor was omitted, its real value varies from 3% to 21% of *d* (see Figure 1c).

## Supplementary Materials

The following are available online at https://www.mdpi.com/1996-1944/13/14/3088/s1. Table S1: Tabularized data for all silica nanoparticle samples, Code S1: Maple code for the calculation of *Q_scat_* and *τ_sp_* using Mie theory.
