# Supplementary material for: “DIY” Silica Nanoparticles: Exploring the Scope of a Simplified Synthetic Procedure and Absorbance-Based Diameter Measurements
Source: Materials (Basel). 2020 Jul 10;13(14):3088. doi: 10.3390/ma13143088 (PMC7412208; doi:10.3390/ma13143088)
Supplement: Supplementary file 1 [file materials-13-03088-s001.pdf]

materials

MDPI

Supplementary materials

"DIY" Silica Nanoparticles: Exploring the Scope of a Simplified Synthetic Procedure and Absorbance-Based Diameter Measurements

Łukasz Tabisz *, Jerzy Stanek and Bogusława Łęska

Faculty of Chemistry, Adam Mickiewicz University in Poznań, Uniwersytetu Poznańskiego 8, 61-614 Poznań, Poland, stanek@amu.edu.pl (J.S.); bogunial@amu.edu.pl (B.Ł.)

* Correspondence: lukasz.tabisz@amu.edu.pl

Table S1. Tabularized data for all silica nanoparticle samples.

Series /	Volume of reagents (mL)			Molar concentration of reagents (mol·L⁻¹)				Product	TEM data (nm)		Calculated data	
sample	TEOS	NH_3	H_2O	TEOS	NH_3	H_2O	EtOH	$[NH_3]\cdot[H_2O]]$	*d*	SD_d	τ_{sp} (cm^{-1})	d_{calc} (nm)
1/1	0.40	0.40	9.20	0.1805	0.5343	3.1700	15.5287	1.6938	63.280	7.930	-	-
1/2	0.40	0.50	9.10	0.1805	0.6679	3.5309	15.3599	2.3583	131.463	12.245	-	-
1/3	0.40	0.60	9.00	0.1805	0.8015	3.8918	15.1911	3.1192	253.835	12.943	-	-
1/4	0.40	0.70	8.90	0.1805	0.9351	4.2526	15.0223	3.9765	266.276	14.552	-	-
1/5	0.40	0.80	8.80	0.1805	1.0686	4.6135	14.8535	4.9302	294.691	17.411	-	-
1/6	0.40	0.90	8.70	0.1805	1.2022	4.9744	14.6848	5.9803	291.014	19.598	-	-
1/7	0.40	1.00	8.60	0.1805	1.3358	5.3352	14.5160	7.1268	299.843	15.334	-	-
1/8	0.40	1.10	8.50	0.1805	1.4694	5.6961	14.3472	8.3697	299.820	16.031	-	-
1/9	0.40	1.20	8.40	0.1805	1.6030	6.0570	14.1784	9.7090	317.531	16.330	-	-
1/10	0.40	1.30	8.30	0.1805	1.7365	6.4178	14.0096	11.1448	325.260	16.752	-	-
1/11	0.40	1.40	8.20	0.1805	1.8701	6.7787	13.8408	12.6769	337.832	11.420	-	-
1/12	0.40	1.50	8.10	0.1805	2.0037	7.1396	13.6720	14.3055	353.191	18.574	-	-
1/13	0.40	1.60	8.00	0.1805	2.1373	7.5004	13.5032	16.0305	373.210	21.221	-	-
1/14	0.40	1.80	7.80	0.1805	2.4044	8.2221	13.1656	19.7697	383.613	20.744	-	-
1/15	0.40	2.00	7.60	0.1805	2.6716	8.9439	12.8281	23.8945	386.761	17.463	-	-
1/16	0.40	2.20	7.40	0.1805	2.9388	9.6656	12.4905	28.4049	395.760	17.181	-	-
1/17	0.40	2.40	7.20	0.1805	3.2059	10.3873	12.1529	33.3010	405.391	14.122	-	-
1/18	0.40	2.60	7.00	0.1805	3.4731	11.1091	11.8153	38.5827	420.410	14.363	-	-
2/1	0.40	0.36	9.24	0.1805	0.4809	1.4470	16.2136	0.6958	13.242	2.750	28.737	51.943
2/2	0.40	0.38	9.22	0.1805	0.5076	1.5225	16.1785	0.7728	19.647	3.122	28.198	51.910
2/3	0.40	0.40	9.20	0.1805	0.5343	1.5981	16.1434	0.8539	25.812	3.528	52.422	52.716
2/4	0.40	0.42	9.18	0.1805	0.5610	1.6737	16.1083	0.9390	27.825	4.043	54.389	52.785
2/5	0.40	0.44	9.16	0.1805	0.5878	1.7493	16.0732	1.0282	33.458	4.646	76.335	53.639
2/6	0.40	0.46	9.14	0.1805	0.6145	1.8249	16.0381	1.1213	40.472	4.938	104.079	54.624
2/7	0.40	0.48	9.12	0.1805	0.6412	1.9005	16.0030	1.2186	44.143	5.658	141.553	55.935
2/8	0.40	0.50	9.10	0.1805	0.6679	1.9761	15.9679	1.3198	47.778	6.292	172.340	57.013
2/9	0.40	0.52	9.08	0.1805	0.6946	2.0517	15.9328	1.4251	53.635	6.969	298.965	61.445
2/10	0.40	0.54	9.06	0.1805	0.7213	2.1273	15.8977	1.5345	61.214	7.524	409.897	65.327
2/11	0.40	0.56	9.04	0.1805	0.7480	2.2029	15.8626	1.6478	67.213	9.311	537.734	69.801
2/12	0.40	0.58	9.02	0.1805	0.7748	2.2784	15.8275	1.7653	72.103	9.919	587.579	71.546
2/13	0.40	0.60	9.00	0.1805	0.8015	2.3540	15.7924	1.8867	81.944	11.922	820.209	79.688
2/14	0.40	0.70	8.90	0.1805	0.9351	2.7320	15.6170	2.5546	101.456	13.419	1273.814	95.564
2/15	0.40	0.75	8.85	0.1805	1.0018	2.9210	15.5292	2.9264	131.319	21.545	2191.105	127.670
2/16	0.40	0.90	8.70	0.1805	1.2022	3.4879	15.2660	4.1932	277.079	10.215	6079.522	263.764
2/17	0.40	1.50	8.10	0.1805	2.0037	5.7556	14.2132	11.5325	348.721	11.889	8590.091	351.634
2/18	0.40	2.40	7.20	0.1805	3.2059	9.1572	12.6340	29.3571	396.782	9.086	8037.985	332.310
3/1	0.40	0.40	9.20	0.1805	0.5343	1.5981	16.1434	0.8539	26.321	3.911	35.928	52.239
3/2	0.40	0.45	9.15	0.1805	0.6011	1.7871	16.0557	1.0742	39.112	4.578	120.566	55.201
3/3	0.40	0.50	9.10	0.1805	0.6679	1.9761	15.9679	1.3198	48.213	6.835	230.532	59.045
3/4	0.40	0.55	9.05	0.1805	0.7347	2.1651	15.8802	1.5906	65.781	9.023	450.968	66.765
3/5	0.40	0.60	9.00	0.1805	0.8015	2.3540	15.7924	1.8867	82.094	12.012	880.776	81.808
3/6	0.40	0.65	8.95	0.1805	0.8683	2.5430	15.7047	2.2080	95.679	12.982	1325.178	97.362
3/7	0.40	0.70	8.90	0.1805	0.9351	2.7320	15.6170	2.5546	114.135	14.452	1804.655	114.144
3/8	0.40	0.75	8.85	0.1805	1.0018	2.9210	15.5292	2.9264	136.078	15.676	2311.671	131.889

Code S1. Maple code for calculation of Q_{scat} and τ_{sp} using Mie theory.

```
> Digits := 24;
> lambda0:=400;       # incident wavelength in nanometers
> eta_p:=w->c1*(-40.596*w^5+93.805*w^4-86.36*w^3+39.876*w^2-9.3768*w+2.3822);
> eta_m:=w->c2*(-26.231*w^5+63.835*w^4-61.969*w^3+30.217*w^2-7.5228*w+2.1481);
# polynomial fits for data from Ref. [39]
> c1:=1.0;  # used to simulate non-ideal material; see Figure 3(a)
> c2:=1.0;  # used to simulate non-ideal medium; see Figure 3(a)
> lambda_m:= lambda0/eta_m((1e-3)*lambda0);
> m:=eta_p(lambda0*1e-3)/eta_m(lambda0*1e-3);
> alpha0:=evalf(Pi/lambda_m);         # from Eq. (6) alpha=Pi/lambda_m*d=alpha0*d
> psi:=(n,z)->sqrt(Pi*z/2)*BesselJ(n+1/2,z);
> psi_p:=(n,z)->D[2](psi)(n,z);
> zeta:=(n,z)->sqrt(Pi*z/2)*(BesselJ(n+1/2,z)+I*(-1)^n*BesselJ(-n-1/2,z));
> zeta_p:=(n,z)->D[2](zeta)(n,z);
> An:=(n,z)->psi_p(n,z)/psi(n,z);
> an:=(n,x,y)->(An(n,y)*psi(n,x)-m*psi_p(n,x))/(An(n,y)*zeta(n,x)-m*zeta_p(n,x));      # Eq. (10) in Ref. [36]
> bn:=(n,x,y)->(m*An(n,y)*psi(n,x)-psi_p(n,x))/(m*An(n,y)*zeta(n,x)-zeta_p(n,x));      # Eq. (11) in Ref. [36]
> nmax:=70;
> Qscat:=(nmax,x,y)-
>2/x^2*add((2*n+1)*(an(n,x,y)*conjugate(an(n,x,y))+bn(n,x,y)*conjugate(bn(n,x,y))),n=1..nmax);
# Eq. (5) in Ref. [36]
> plot(Qscat(nmax,alpha0*d,alpha0*m*d), d=10..5000);
> tau_sp:=(nmax,x,y,d)->(3/(2*d))*Qscat(nmax,x,y);
> plot(tau_sp(nmax,alpha0*d,alpha0*m*d,d),d=0..350); # by default tau_sp values will be calculated in nm^-
1
```

© 2020 by the authors. Submitted for possible open access publication under the terms and conditions of the Creative Commons Attribution (CC BY) license (http://creativecommons.org/licenses/by/4.0/).
